# Transcriptome Wide Identification and Validation of Calcium Sensor Gene Family in the Developing Spikes of Finger Millet Genotypes for Elucidating Its Role in Grain Calcium Accumulation

**DOI:** 10.1371/journal.pone.0103963

**Published:** 2014-08-26

**Authors:** Uma M. Singh, Muktesh Chandra, Shailesh C. Shankhdhar, Anil Kumar

**Affiliations:** 1 Department of Molecular Biology and Genetic Engineering, Govind Ballabh Pant University of Agriculture and Technology, Pantnagar, Uttarakhand, India; 2 Department of Plant Physiology, Govind Ballabh Pant University of Agriculture and Technology, Pantnagar, Uttarakhand, India; National Institute of Plant Genome Research, India

## Abstract

**Background:**

In finger millet, calcium is one of the important and abundant mineral elements. The molecular mechanisms involved in calcium accumulation in plants remains poorly understood. Transcriptome sequencing of genetically diverse genotypes of finger millet differing in grain calcium content will help in understanding the trait.

**Principal Finding:**

In this study, the transcriptome sequencing of spike tissues of two genotypes of finger millet differing in their grain calcium content, were performed for the first time. Out of 109,218 contigs, 78 contigs in case of GP-1 (Low Ca genotype) and out of 120,130 contigs 76 contigs in case of GP-45 (High Ca genotype), were identified as calcium sensor genes. Through *in silico* analysis all 82 unique calcium sensor genes were classified into eight calcium sensor gene family *viz.*, CaM & CaMLs, CBLs, CIPKs, CRKs, PEPRKs, CDPKs, CaMKs and CCaMK. Out of 82 genes, 12 were found diverse from the rice orthologs. The differential expression analysis on the basis of FPKM value resulted in 24 genes highly expressed in GP-45 and 11 genes highly expressed in GP-1. Ten of the 35 differentially expressed genes could be assigned to three documented pathways involved mainly in stress responses. Furthermore, validation of selected calcium sensor responder genes was also performed by qPCR, in developing spikes of both genotypes grown on different concentration of exogenous calcium.

**Conclusion:**

Through *de novo* transcriptome data assembly and analysis, we reported the comprehensive identification and functional characterization of calcium sensor gene family. The calcium sensor gene family identified and characterized in this study will facilitate in understanding the molecular basis of calcium accumulation and development of calcium biofortified crops. Moreover, this study also supported that identification and characterization of gene family through Illumina paired-end sequencing is a potential tool for generating the genomic information of gene family in non-model species.

## Introduction

Calcium is an important essential element, acts as secondary messenger in various biological processes. Its deficiency causes low bone density, osteoporosis, colon cancer etc [1]. Milk is the major source of calcium for humans in the world. Unavailability of recommended amount of milk in poor population and lactose intolerance may lead to calcium deficiency. Plant based calcium could serve as an alternate source for calcium. Since, daily consumed cereals are poor in calcium, biofortification of these cereals for calcium and understanding molecular basis of its accumulation is in need. Finger millet, rich in calcium and contains about 5–30 times higher calcium in contrast to rice and wheat [2]. In general, plants absorb calcium through roots and deliver it to shoots through the xylem stream [3]. The process of calcium uptake and transport is genetically and epigenetically determined [4]. Earlier studies suggested the roles of various calcium transporters in the accumulation of calcium in plants [5] but the role of calcium sensor genes in regulating calcium transportation has not been reported till date.

In plants, calcium sensor proteins are categorized into two groups *i.e.*, calcium sensor relay and calcium sensor responder [6]. The calcium relay proteins bind calcium and affect their target protein because they themselves do not have enzymatic activity [6]. In contrast, the calcium sensor responder proteins bind calcium and a change in conformation takes place and hence modulates their own activity by intra-molecular interaction. As per literature, two types of calcium sensor protein *viz.*, Calmodulin (CaM) and Calciuneurin B-like protein (CBL) have been reported likewise calcium responders have been classified into six types *viz.*, (i) Ca^2+^ dependent and CaM independent protein kinases (CDPKs); (ii) SOS3/CBL interacting protein kinases (SIPKs/CIPKs); (iii) CaM dependent protein kinases (CaMKs); (iv) Ca^2+^/CaM dependent protein kinases (CCaMKs); (v) CDPK related protein kinases (CRKs); (vi) phosphoenolpyruvate (PEP) carboxylase kinase-related kinases (PEPRKs) [7]. The role of the calcium sensor genes are reported in the regulation of calcium transporter proteins [9]. Like calcium dependent protein kinases-1 (*CDPK-1*) was reported in regulation of PM type Ca^2+^-ATPase2 in Arabidopsis by phosphorylation within their N-terminal regulatory domain [8]. However, the exact nature and role of calcium sensor genes in seed calcium accumulation in finger millet requires detailed investigation.

Earlier some studies have been conducted for understanding the role of these genes in seed calcium accumulation, no fruitful inference could be made due to limited genetic information of finger millet. Considering the problems faced by earlier groups, in present study transcriptomics approach was used to characterize calcium sensor gene family from developing spikes of finger millet. This work will represent the first exhaustive analysis for calcium sensor genes in cereal crops. The characterisation, identification, classification, phylogeny and pathway analysis are therefore important steps in understanding the role of calcium sensor genes in grain calcium accumulation that may further highlight our knowledge on plant calcium signaling. The production of specialised cDNA from spikes was used to determine the expression patterns of all calcium sensor genes and highly expressed genes in contrasting genotypes were validated using qPCR analysis.

## Materials and Methods

### Tissue collection and RNA isolation

Finger millet genotypes *i.e.*, GP-1 (200 mg/100 g seed) and GP-45 (400 mg/100 g seed) differing in their grain calcium content were obtained from Rani Chauri Hill Campus, G.B. Pant University of Agriculture and technology, Pantnagar (Uttarakhand) India. The spike/panicle samples were selected at four stages *viz.*, S1 (spike emergence); S2 (pollination stage); S3 (dough stage) and S4 (maturation stage) on the basis of its morphology along with stages of ovary and anther. RNA was isolated by using total RNA isolation protocol [7]. Total RNA was treated with RNase- free DNase I (Fermentas, Germany) for 30 min at 37°C to remove residual DNA. The quality of RNA samples was checked both by agarose gel electrophoresis and RNA integrity number (RIN) value estimation. The appearance of two prominent bands of 16S and 28S rRNA in RNA sample and the RIN value of both RNA sample was recorded to be 8, which confirmed good quality and integrity of RNA samples 8.

### Preparation of cDNA and transcriptome sequencing

Equal amount of RNA from developing spikes collected at all four stages (S1, S2, S3, S4) of both genotypes (GP-1 & GP-45) were mixed separately for subsequent analysis. Pooled RNA were used to purify poly (A) mRNA using Oligotex mRNA midi prep kit (Qiagen, Germany) followed by fragmentation into 200–500 bp pieces using divalent cations at 94°C for 5 min. The cleaved RNA fragments were copied into first strand cDNA using Superscript II reverse transcriptase (Life Technologies, Inc.) and random primers. After second strand cDNA synthesis, fragments were end repaired, a-tailed and indexed adapters were ligated. The products were purified and enriched with PCR to create the final cDNA library. The cDNA libraries were used for 2×100 bp paired-end sequencing on a single lane of the Illumina HiSeq 2000 (Genomics Core, UZ Leuven, Belgium). After sequencing, the samples were demultiplexed and the indexed adapter sequences were trimmed using the CASAVA v1.8.2 software (Illumina, Inc.). The sequencing and assembly was done by commercial sequencing service provider (NexGenBio, New Delhi, India). A total of 6.54 GB (Gigabyte) and 7.31 GB sequence data were generated from the GP-1 and GP-45 spikes respectively.

### RNA-Seq data filter and *De novo* assembly

The customer Perl script (CONDETRI: http://code.google.com/p/condetri) with parameters (-hq = 20 -lq = 10 -frac = 0.8 -lfrac = 0.1 -minlen = 50 -mh = 5 -ml = 5 -sc = 64) was used to remove the sequencing adaptor and low quality reads. *De novo* assembly of high quality reads was done by Trinity assembler to generate a non-redundant set of transcripts using one k-mer length (25-mer) and group_pairs_distance = 250, path_reinforcement_ distance = 70, min_glue = 2, min_kmer_cov = 2 keeping other default parameters [10]. The high quality read obtained after sequencing were assembled *de novo* using the Trinity program [15], which produced 109,218 contigs, with an N50 of 1191 bp in GP-1 and 120,130 contigs, with an N50 of 1450 bp in GP-45 genotype. GP-1 and GP-45 transcripts were deposited at NCBI/Gene Bank as the TSA accession SRR1151079 and SRR1151080 respectively and used for further analysis.

### Identification and annotation of calcium sensor gene family from finger millet transcriptome data

To identify Calcium sensor genes from transcriptome of both GP-1 and GP-45 genotypes, Coding DNA Sequence (CDS) of rice calcium sensor genes *viz.*, CaM, CMLs, CBLs, CDPKs, CIPKs, CaMKs, CCaMK, CRKs and PEPRKs were retrieved from various sequence databases. Transcripts of both GP-1 and GP-45 genotypes were used to make offline database separately. Rice calcium sensor genes were searched as query sequences from nucleotide sequence of the full transcriptome data of both genotypes using UGENE (UGENE, UniPro, Russia). Afterwards, the selected calcium sensor genes sequences were performed a BLASTn search.

Each gene gave around 100–200 hits in the contigs, hence an excel sheet of all calcium sensor was made which was a huge file of data influx. Then the filters were applied to specify the data according to the e-value (<1) and large amount of data redundancy was thus removed. Finally, the truncated data was cross checked, with database by using the blast search tool BLASTx and the hits with minimum expected value, maximum identity with maximum query coverage were selected.

### Re-assembly of retrieved sequences and ORF identification

Hence, contigs verified were used to select the best non redundant contig using SeqMan Pro gene analysis package (DNASTAR Inc., Madison, WI, USA). An ORF finder (http://www.ncbi.nlm.nih.gov/projects/gorf/) online tool was used for identification of open reading frame (ORF) in annotated sequences.

### Classification of calcium sensor genes

The calcium sensor genes retrieved from finger millet transcriptome and rice genome database were aligned and evolutionary analyses using Neighbor-Joining (NJ) algorithm were constructed using MEGA6 (http://www.megasoftware.net) [11]. Following parameters were set during construction of phylogenetic tree *viz.*, substitution, poisson model, complete deletion, replication, bootstrap analysis with 1,000 replicates. The percentage similarities of finger millet sequence with rice sequence were also checked through EBI online software (https://www.ebi.ac.uk/Tools/psa/emboss_needle/). Structural and functional verification of each gene was also predicted using ScanProsite (release 20.83) (http://prosite.expasy.org/scanprosite/) and SMART tools (http://smart.embl-heidelberg.de/).

### Properties of calcium sensor genes

Analysis of the functional and physiochemical properties of each protein was done by Protein Identification and Analysis Tools on ExPASy Server (http://web.expasy.org/protparam/) and domains were analysed using ScanProsite detection of PROSITE signature matches and ProRule-associated functional and structural residues in proteins (http://prosite.expasy.org/). Prediction of subcellular localization was done by using Target P1.1 server (http://www.cbs.dtu.dk/services/TargetP/). The molecular weight of the protein and its isoelectric point (pI) were estimated by using online expasy server (http://web.expasy.org/compute_pi/). A well tabulated data sheet was prepared comprising the physiochemical properties such as GRAVY, instability index, number of EF hands and their sub-cellular localization.

### Transcriptome based expression analysis of identified calcium sensor genes

To determine the differentially expressed genes between GP-1 and GP-45, in present analysis, the sequencing reads were mapped to each gene and presented in **F**ragments **P**er **K**ilobase of exon per **M**illion fragments mapped (FPKM). In both GP-1 and GP-45 transcriptome data FPKM value of transcripts were compared. The gene expression were measured as normalized expected fragments, allowing for measurement of read counts from platforms that produce one or more reads per single source molecules [12]. Pathway analysis was also performed using Kyoto Encyclopaedia of Genes and Genomes (KEGG) (http://www.genome.jp/kegg/).

### Exogenous calcium application and calcium analysis

Seeds of GP-1 and GP-45 genotypes were sown in kharif season of 2011–12 in the pots. The seedlings were transplanted and grown in individual pots (25 cm upper diameter, 17 cm lower diameter, 25 cm height) filled with acid washed sand and placed in the experimental polyhouse. The plants were provided with half strength of macro and micronutrient in a modified Hoagland nutrient solution [13] containing different concentrations of calcium (0.1 mM, 5.0 mM, 10 mM and 20 mM) in the form of Ca(NO_3_)_2_.4H_2_O. The nutrient solution (pH 5.5–6.0) was renewed every three days interval after the sand had been rinsed with distilled water. The experimental design was completely randomized with four treatments, arranged in individual pot with nine pots per treatment, each replicated three times. The spikes were sampled at each stage of spike development (S1, S2, S3 and S4) from each treatment. The samples for calcium estimation by atomic absorption spectroscopy (AAS) (SensAA GBC Scientific Equipment, USA), were prepared by wet digestion method [14]. To detect differences between treatments, analysis of variance (ANOVA) was performed using SPSS 21.0 (SPSS Inc.,Chicago, IL, USA).

### RNA isolation and qPCR analysis

Total RNA was isolated from the spike collected at each four stages according to protocol described by Ghawana *et al.* [15]. Isolated RNA was treated with RNase-free DNase I for 30 min at 37°C to remove residual DNA. cDNA was prepared from the purified RNA using Revert Aid H-minus reverse transcriptase cDNA synthesis kit (Fermentas, Germany). Quantitative real-time PCR (qPCR) was used to identify the expression patterns of selected calcium sensor genes in different stages of spikes of both genotypes grown at different concentration of exogenous calcium. The tubulin gene (CX265249) was used as an internal control to normalize the expression level of the target gene. Real-time PCR was performed in the reaction volume of 20 µl containing 2.5× Real Master Mix SYBR ROX/20× SYBR solution, 100 nM of each forward and reverse primers and 100 ng of cDNA. All samples were amplified in triplicate and the mean and standard error values were calculated. Relative expression of all genes were calculated the by _ΔΔ_CT method.

## Results

### Identification of calcium sensor genes

The rice calcium sensor genes were used as reference for identification of their homologues in finger millet transcriptome. Out of 109,218 contigs (in GP-1) and 120,130 contigs (in GP-45), a total of 138 and 137 contigs respectively representing putative calcium sensor and calcium sensor like protein, were filtered. Only hits with e value of <1.0 were considered for further analysis. The sequence with e value <1.0 were reassembled with SeqMan aligner to reduce redundancy and obtaining maximum length of ORF. The contig sequences which are repeated more than one time were used for open reading frame (ORF) selection. The online tool ORF finder allowed identification of ORFs encoding putative calcium sensor protein in finger millet. Structural and functional verification of calcium binding and related domain were carried out. Finally, we demonstrated 80 and 78 non redundant calcium sensor genes in GP-1 and GP-45 respectively. It includes 17 ORFs encoding for CDPKs, 21 for CIPKs, 2 for CaMKs, 1 for CCaMKs, 2 for PEPRKs, 4 for CRKs, 9 for CBLs and 24 for CaM and CaML proteins in GP-1 transcriptome. Similarly, 16 ORFs encoding for CDPKs, 22 for CIPKs, 2 for CaMK, 1 for CCaMK, 2 for PEPRKs, 4 for CRKs, 8 for CBLs and 23 for CaM and CaML proteins were identified in GP-45 transcriptome.

### Designating the analyzed sequences as calcium sensor genes

All calcium sensors were classified into eight group from A to H (in Phylogenetic tree) *viz.*, CaM and CaML, PEPRK, CIPK, CRK, CaMK, CDPK, CCaMK and CBL genes on the basis of sequence features, identity, phylogenetic study and were designated according to the name of their rice homologues. The first initial of genus and species name were used in the naming of all finger millet sequences (**Table 1**).The group A contained sequences of CaM & CaML gene, group B contained sequences of PEPRK gene, group C contained sequences of CIPK gene, group D contained sequences of CRK gene, group E contained sequences of CaMK gene, group F contained sequences of CDPK gene, group G contained sequences of CCaMK gene and group H contained sequences of CBL gene. The genes falling in same group is interpreted as these genes may be diverged from the same ancestral gene and showing varied function though still maintaining high structural homology.

**Table 1:** List of finger millet Calcium sensor genes and its similarity with its rice orthologs.

S.No.	Ca sensor gene (Oryza)	Accession no. (Oryza)	% identity (Oryza vs Eleusine)
1	OsCaM1	LOC_Os03g20370	EcCaM1 (35.9%)
2	OsCaML1	LOC_Os01g59530	EcCaML1 (71.8%)
3	OsCaML2	LOC_Os11g03980	EcCaML2 (87.4%)
4	OsCaML4	LOC_Os03g53200	EcCaML4 (92.9%)
5	OsCaML5	LOC_Os12g41110	EcCaML5 (80.0%)
6	OsCaML8	LOC_Os10g25010	EcCaML8 (90.8%)
7	OsCaML9	LOC_Os05g41200	EcCaML9 (73.2%)
8	OsCaML10	LOC_Os01g72100	EcCaML10 (87.0%)
9	OsCaML11	LOC_Os01g32120	EcCaM11 (79.9%)
10	OsCaML14	LOC_Os05g50180	EcCaM14 (92.4%)
11	OsCaML17	LOC_Os02g39380	EcCaM17 (57.2%)
12	OsCaML18	LOC_Os05g13580	EcCaML18 (81.1%)
13	OsCaML22	LOC_Os04g41540	EcCaML22 (68.2%)
14	OsCaML23	LOC_Os01g72540	EcCaML23 (75.2%)
15	OsCaML24	Os07g0681400	EcCaML24 (82.6%)
16	OsCaML27	LOC_Os03g21380	EcCaML27 (89.6%)
17	OsCaML28	LOC_Os12g12730	EcCaML28 (83.0%)
18	OsCaML29	LOC_Os06g47640	EcCaML29 (75.5%)
19	OsCaML30	LOC_Os06g07560	EcCaML30 (72.2%)
20	OsCaML31	LOC_Os01g72530	EcCaML31 (86.2%)
21			EcCaML34
22			EcCaML35
23			EcCaML36
24			EcCaML37
25			EcCaML38
26			EcCaML39
27	OsCRK2	BAD54109.1	EcCRK2 (88.1%)
28	OsCRK3	BAC79879.1	EcCRK3 (96.7%)
29	OsCRK5	AAK84452.1	EcCRK5 (93.1%)
30	OsCaMK1	AF368282.1	EcCaMK1 (85.6%)
31	OsCaMK	BAC16472.1	EcCaMK2 (90.9%)
32	OsPEPRK2	BAD17519.1	EcPEPRK2 (72.5%)
33			EcPEPRK3 (84.2%)
34	OsCCaMK1	AAT77292.1	EcCCaMK1 (95.5%)
35	OsCIPK2	AK072868	EcCIPK10 (87.7%)
36	OsCIPK4	Os12g41090	EcCIPK4 (68.9%)
37	OsCIPK5	AK065589	EcCIPK8 (87.7%)
38	OsCIPK6	Os08g34240	EcCIPK13 (80.4%)
39	OsCIPK7	AK111510	EcCIPK18 (79.6%)
40	OsCIPK8	AK120431	EcCIPK3 (90.1%)
41	OsCIPK9	OJ1015F07.8	EcCIPK4 (85.9%)
42	OsCIPK10	AK066541	EcCIPK9 (77.2%)
43	OsCIPK11	AK103032	EcCIPK2 (77.9%)
44	OsCIPK14	Os12g02200	EcCIPK7 (66.7%)
45	OsCIPK16	AK061220	EcCIPK12 (75.0%)
46	OsCIPK17	AK100498	EcCIPK11 (45.2%)
47	OsCIPK18	AK101355	EcCIPK6 (88.4%)
48	OsCIPK19	AK069486	EcCIPK14 (60.1%)
49	OsCIPK21	AK107137	EcCIPK16 (81.6%)
50	OsCIPK23	ACD76983.1	EcCIPK23 (98.0%)
51	OsCIPK24	AK102270	EcCIPK11 (88.4%)
52	OsCIPK25	AK065374	EcCIPK19 (61.2%)
53	OsCIPK28	A3B529.2	EcCIPK28 (34.0%)
54	OsCIPK29	AK111746	EcCIPK17 (76.6%)
55			EcCIPK30
56			EcCIPK31
57			EcCIPK32
58			EcCIPK33
59	OsCDPK1	LOC_Os01g43410.1	EcCDPK2 (87.8%)
60	OsCDPK3	LOC_Os01g61590.1	EcCDPK18 (96.3%)
61	OsCDPK4	AK060738.1	EcCDPK14 (94.9%)
62	OsCDPK6	LOC_Os02g58520.1	EcCDPK16 (87.0%)
63	OsCDPK7	LOC_Os03g03660.2	EcCDPK9 (87.8%)
64	OsCDPK8	LOC_Os03g59390.1	EcCDPK11 (89.8%)
65	OsCDPK12	Os04t0584600	EcCDPK5 (89.9%)
66	OsCDPK13	AK061881	EcCDPK1 (79.1%)
67	OsCDPK14	LOC_Os05g41270.1	EcCDPK7 (77.8%)
68	OsCDPK17	Os07g0161600	EcCDPK12 (58.0%)
69	OsCDPK19	AP003954	EcCDPK3 (94.0%)
70	OsCDPK20	AP003866	EcCDPK8 (89.8%)
71	OsCDPK24	Os11g0171500	EcCDPK6 (88.6%)
72	OsCDPK25	Os11g0136600	EcCDPK31 (90.9%)
73	OsCDPK29	LOC_Os12g12860.1	EcCDPK29 (87.8%)
74	OsCBL1	ABA54176.1	EcCBL1 (96.7%)
75	OsCBL2	ABA54177.1	EcCBL2 (98.7%)
76	OsCBL3	ABA54178.1	EcCBL3 (95.4%)
77	OsCBL4	ABA54179.1	EcCBL4 (86.4%)
78	OsCBL5	ABA54180.1	EcCBL5 (90.2%)
79	OsCBL6	ABA54181.1	EcCBL6 (62.0%)
80	OsCBL7	ABA54182.1	EcCBL7 (87.3%)
81	OsCBL9	ABA54184.1	EcCBL9 (87.9%)
82	OsCBL10	ABA54185.1	EcCBL10 (84.2%)

### Structural and functional analysis of calcium sensor genes in finger millet

#### Domain analysis of calcium sensor genes

The domain analysis revealed that most of CaM and CaML genes contained 4 EF-hand, however, 5 contained 3 EF-hand and another 5 contained 2 EF-hand. The PEPRK gene family comprises of only PKC (Protein kinase) domain in finger millet. The CIPK family in finger millet have no EF hand at the carboxyl terminal, but have auto inhibitory domain also known as NAF domain. CRK genes in finger millet possess only single kinase and single EF-hand with an exception of *EcCRK3* having both kinase as well as an EF-hand while *EcCRK2*, 5 and 7 possess only kinase domain. Similar to rice CaMK, finger millet CaMK also contains one kinase and one EF-hand.

Domain analysis revealed that a conserved protein kinase domain was found in EcCDPKs along with four EF-hand domains, with an exception of *EcCDPK6* which only contain four EF- hand domains and protein kinase domain was absent (**Table 2**). In finger millet CCaMKs have the same general structure of CDPKs, but they have calcium-binding domains with three EF-hands. Structure of CBL are similar to CaM and CaML gene, however unlike CaM, CBL possess only 3 EF-hand, with exception of *EcCBL3* which possess only 2 EF-hand.

**Table 2:** List of domain, sub-cellular localization and instability index analysis of finger millet calcium sensor.

S.No	Gene name	Domain	GRAVY	Instability index	Stability	Localization
**A. CaM and CaML proteins**						
1	EcCaM1	4 EF-hand	−0.602	23.23	stable	-
2	EcCaML2	4 EF-hand	−0.613	38.96	stable	-
3	EcCaML4	4 EF-hand	−0.449	28.05	stable	-
4	EcCaML5	2 EF-hand	−0.946	33.75	stable	-
5	EcCaML8	4 EF-hand	−0.721	20.32	stable	-
6	EcCaML9	3 EF-hand	−0.531	38.70	stable	-
7	EcCaML10	4 EF-hand	−0.226	60.77	unstable	C
8	EcCaML11	4 EF-hand	−0.226	39.16	stable	-
9	EcCaML14	4 EF-hand	0.120	17.34	stable	M
10	EcCaML18	4 EF-hand	−0.332	30.12	stable	-
11	EcCaML22	4 EF-hand	−0.572	61.46	unstable	C
12	EcCaML23	4 EF-hand	−0.370	41.43	unstable	-
13	EcCaML24	3 EF-hand	−0.254	20.22	stable	C
14	EcCaML27	3 EF-hand	−0.210	29.80	stable	C
15	EcCaML28	4 EF-hand	−0.693	19.61	stable	
16	EcCaML29	3 EF-hand	0.128	33.17	stable	-
17	EcCaML30	2 EF-hand	−0.369	51.99	unstable	S
18	EcCaML31	3 EF-hand	−0.168	27.03	stable	-
19	EcCaML34	2 EF-hand	−0.0397	68.38	unstable	C
20	EcCaML35	2 EF-hand	−0.179	66.71	unstable	C
21	EcCaML36	3 EF-hand	−0.448	46.78	unstable	C
22	EcCaML37	3 EF-hand	−0.954	30.71	stable	M
23	EcCaML38	3 EF-hand	−0.076	24.45	stable	-
24	EcCaML39	4 EF-hand	−0.642	36.45	stable	M
**B. EcPEPRK proteins**						
1	EcPEPRK2	PKC	0.297	54.25	unstable	S
2	EcPEPRK3	PKC	−0.53	45.09	unstable	-
**C. EcCRK proteins**						
1	EcCRK2	PKC	−0.395	58.56	unstable	-
2	EcCRK3	PKC	−0.161	40.17	unstable	-
3	EcCRK5	PKC	−0.248	49.56	unstable	S
**D. EcCaMK proteins**						
1	EcCaMK1	PKC, 1 EF hand	−0.245	44.63	unstable	-
2	EcCaMK2	PKC, 1 EF hand	−0.259	43.88	unstable	-
**E. EcCCaMK protein**						
1	EcCCaMK1	PKC, 3 EF hand	−0.215	46.23	unstable	-
**F. EcCBL proteins**						
1	EcCBL1	3 EF hand	−0.241	27.76	stable	-
2	EcCBL2	3 EF hand	−0.216	32.51	stable	S
3	EcCBL3	2 EF hand	−0.017	42.23	unstable	-
4	EcCBL4	3 EF hand	−0.260	46.04	unstable	-
5	EcCBL5	3 EF hand	−0.317	50.54	unstable	-
6	EcCBL6	3 EF hand	−0.331	37.08	stable	M
7	EcCBL7	3 EF hand	−0.229	44.93	unstable	M
8	EcCBL9	3 EF hand	−0.220	35.66	stable	-
9	EcCBL10	3 EF hand	−0.137	44.08	unstable	C
**G. EcCDPK proteins**						
1	EcCDPK1	PKC, 4 EF hand	−0.524	45.98	unstable	M
2	EcCDPK3	PKC, 4 EF hand	−0.467	38.40	stable	-
3	EcCDPK4	PKC, 4 EF hand	−0.387	45.59	unstable	-
4	EcCDPK6	4 EF hand	−0.350	30.95	stable	-
5	EcCDPK7	PKC, 4 EF hand	−0.322	42.97	unstable	-
6	EcCDPK8	PKC, 4 EF hand	−0.499	36.43	stable	-
7	EcCDPK12	PKC, 4 EF hand	−0.330	33.95	stable	-
8	EcCDPK13	PKC, 4 EF hand	−0.342	38.54	stable	C
9	EcCDPK14	PKC, 4 EF hand	−0.485	42.10	unstable	-
10	EcCDPK17	PKC, 4 EF hand	−0.299	43.63	unstable	C
11	EcCDPK19	PKC, 4 EF hand	−0.443	42.65	unstable	-
12	EcCDPK20	PKC, 4 EF hand	−0.487	34.31	stable	-
13	EcCDPK24	PKC, 4 EF hand	−0.307	35.31	stable	C
14	EcCDPK25	PKC, 4 EF hand	−0.403	22.97	stable	-
15	EcCDPK29	PKC, 4 EF hand	−0.405	36.32	stable	-
**H. EcCIPK proteins**						
1	EcCIPK2	PKC, NAF	−0.401	30.13	stable	-
2	EcCIPK4	NAF	−0.562	44.22	unstable	M
3	EcCIPK5	PKC, NAF	−0.452	33.39	stable	M
4	EcCIPK6	PKC, NAF	−0.133	31.50	stable	-
5	EcCIPK7	PKC, NAF	−0.146	56.39	unstable	M
6	EcCIPK8	PKC, NAF	−0.289	37.40	stable	-
7	EcCIPK9	PKC, NAF	−0.392	34.93	stable	-
8	EcCIPK10	PKC, NAF	−0.385	34.20	stable	-
9	EcCIPK11	PKC, NAF	−0.494	49.27	unstable	M
10	EcCIPK14	PKC, NAF	−0.370	47.32	unstable	C
11	EcCIPK16	PKC, NAF	0.080	36.17	stable	S
12	EcCIPK18	PKC, NAF	−0.360	30.75	stable	-
13	EcCIPK19	PKC, NAF	−0.384	37.33	stable	C
14	EcCIPK21	PKC, NAF	−0.328	41.36	unstable	-
15	EcCIPK23	PKC, NAF	−0.394	37.56	unstable	-
16	EcCIPK24	PKC, NAF	−0.230	33.20	stable	M
17	EcCIPK25	PKC, NAF	−0.228	49.45	unstable	-
18	EcCIPK28	PKC, NAF	−0.365	37.03	unstable	-
19	EcCIPK29	PKC, NAF	−0.123	40.93	unstable	-
20	EcCIPK31	PKC, NAF	−0.546	48.35	unstable	M
21	EcCIPK32	PKC, NAF	−0.460	39.34	stable	M
22	EcCIPK33	PKC, NAF	−0.373	50.28	unstable	-
23	EcCIPK34	PKC, NAF	−0.329	41.66	unstable	-

C Chloroplast, i.e. the sequence contains cTP, a chloroplast transit peptide;

M Mitochondrion, i.e. the sequence contains mTP, a mitochondrial targeting peptide;

S Secretory pathway, i.e. the sequence contains SP, a signal peptide;

_ any other location.

#### Motif analysis of calcium sensor genes

In case of PEPRK, CRK, CaMK and CCaMK the motif 2, 3 and 7 were common for each gene. The PEPRK and CCaMK sequences contain only five motifs, but CRK and CaMK gene sequences contain the entire ten motifs. Most of CaM and CaML comprises of four motifs out of ten rest six were not uniformly present. In case of CIPK, out of ten motifs nearly all motifs are present in each CIPK genes, except in case of *EcCIPK18*, where motif 9 was doubled and motif 10 was absent. Out of ten motifs, all motifs were found in most of CDPKs of finger millet and rice. Motif 4 was absent in case of *EcCDPK4* gene. Multilevel consensus sequence analysis for all 10 motifs of finger millet showed common sequences among gene family (**Table 3**).

**Table 3:** Multilevel consensus sequences for the MEME defined motifs of members of different calcium sensor genes.

S.No	E-value	Motif length	Motifs
**CaM and CaML**			
1	1.4e-652	33	LREAFRVFDKDQNGYISAAELRHVMTNLGEKCT
2	3.1e-612	24	VEECQQMICNVDRDGDGQINYHEF
3	2.4e-468	21	LRRVFRLFDKNGDGCITTKEL
4	5.9e-317	41	QNPTEDELQAMINEVDTNGNGCIDFHEFVNLYCRKMKDHDH
5	2.70E-45	50	AQALDYHGLSADRAGLTATVGAYIPWGAAGLRFEDFESLHRALGDALFGP
6	7.10E-40	21	SLSKKPSPSFRLRNGSLNVVR
7	4.70E-23	11	KCMMQGITVWG
8	2.10E-10	8	CTVMRSLG
9	2.50E-04	49	MHGIAPSSPDRSPQYPSCAPIKGRRRSRLFHRCTAQPKCAKNDYSLCCS
10	1.70E-01	11	HHQLTQQQIKE
**CRK**			
11	2.2e-368	41	KILKSLSGHNNLVQFYDACEDEDNVYIVMELCEGGELLDRI
12	6.5e-379	35	RCYSTEADMWSIGVITYILLCGSRPFWARTESGIF
13	3.30E-273	50	LKAEPNFNEHPWPTISPEAKDFVKRMLNKDYRKRMTAAQALCHPWIRNYQ
14	6.90E-235	50	ARGGKYSEEDAKCVMVQILSVVSFCHLQGVVHRDLKPENFLFTTKDENSP
15	3.90E-268	50	GEEVGRGHFGYTCSAKAKKGEHKGQDVAVKVIPKAKMTTAIAIEDVRREV
16	3.20E-246	50	FVNTLCNLQYRKMDFEEFCAAAISVYQMEALDTWEQHARTAYEYFEKEGN
17	7.40E-216	29	DFGLSDFVKPDERLNDIVGSAYYVAPEVL
18	1.20E-184	41	EQGMNPSVPLHVVLQDWIRHSDGKLSFLGFIKLLHGMSMRS
19	2.30E-172	41	NSSVASTPARGGFKRPFPPPSPAKHIRALLARRHGSVKPNE
20	1.30E-143	41	DMIIYKLMKAYIRSSSLRKAALRALSKTLTTDQLFYLKEQF
**CIPK**			
21	2.3e-1717	50	EDEARRYFQQLINAVDYCHSRGVYHRDLKPENLLLDENGNLKVSDFGLSA
22	8.1e-1689	27	YDGAKADIWSCGVILYVLMAGYLPFHD
23	1.4e-1212	50	GMMEQIKREISTMKLVRHPNVVQLHEVMATKTKIYFVMEYVTGGELFNKI
24	2.7e-726	30	RLIRRILDPNPMTRITIAEIMEHPWFKKGY
25	1.1e-722	41	KEGRKGVLAIDAEIFEVTPSFHMVELKKTNGDTLEYQQFCN
26	2.9e-566	29	AQCWRQDGLLHTTCGTPNYVAPEVINNKG
27	3.4e-458	29	RYELGRTLGQGTFAKVYHARNTETGQSVA
28	1.70E-253	21	NLMTMYRKICKAEFRCPPWFS
29	1.70E-278	17	MSQGFDLSGMFEEEQEY
30	5.40E-227	41	KRETRFTSQCPPQEIFSKIEEIATPMGFQVQKQNYKMKLMG
**CDPK**			
31	1.6e-1779	50	DIVGSPYYMAPEVLKRNYGPEADVWSAGVILYILLCGVPPFWAETEQGIF
32	2.2e-1374	50	CAGGELFDRIIARGHYTERAAAQLCRTIVGVVHMCHSMGVMHRDLKPENF
33	9.4e-1369	50	VLSRMKQFSAMNKFKKMALRVIAENLSEEEIAGLKEMFKMMDTDNSGTIT
34	3.6e-1363	50	NNGTIDYGEFITATMHMNKMEREEHLYKAFQYFDKDGSGYITIDELQQAC
35	1.4e-1151	50	QAILRGHIDFKSEPWPSISESAKDLVRKMLNPDPKKRLTAHQVLCHPWIC
36	4.3e-1061	50	LGQGQFGTTYLCTHRATGCRYACKSISKRKLVTPEDVEDVRREIQIMHHL
37	2.5e-747	29	IKDIIQEVDQDNDGRIDYQEFVAMMQKGN
38	7.9e-623	21	LFANKKEDSPLKAIDFGLSVF
39	4.7e-517	29	EELKEGLRKYGSNLSESEIQQLMEAADID
40	3.5e-397	21	NIVSIRGAYEDAHAVHLVMEL
**CBL**			
41	1.0e-592	50	NGVIGFGEFVRALSVFHPNAPLEEKIDFAFRLYDLRQTGYIERQEVKQMV
42	2.3e-552	50	DKTFEQADTNHDGKIDQEEWRNFVLRHPSLLKNMTLPYLKDITTTFPSFV
43	4.0e-414	50	ALYELYKKISCSVIDDGLIHKEEFQLALFRTSKKENLFADRVFDLFDTKH
44	3.10E-142	29	MGCCCSKQFKQPPGYEDPQVLARETVFSV
45	1.10E-84	15	ESGMNLSDDIVEAII
46	2.20E-68	50	MDPSSSSNAFGSRSSLTLGELACAALFPVLAILDAVIFATARCFQKSPPR
47	4.60E-16	21	RRPSGGNLFVDRVFDLFDQKR
48	5.90E-13	6	NSQVDD
49	1.80E-09	11	MVQCLDGVRQL
50	2.00E-01	8	CFTVNEVE

#### Sub-Cellular localization, stability and instability index in calcium sensor genes

Based on the information available in the database it was inferred that a total of 13 calcium sensor genes were chloroplast localized contained cTP (a chloroplast transit peptide), 13 were mitochondria localized contained mTP (a mitochondrial targeting peptide) and 5 were present on secretary pathways contained ‘SP’ (a signal peptide site) and hence were predicted to be located at chloroplast, mitochondria and at the secretary pathway respectively (**Table 2**). Other calcium sensor sequence residing sites could not be predicted due to limitation of the information available in the database. The stability parameters of calcium sensors suggested that 40 calcium sensor genes were found to be stable and remaining 32 calcium sensor genes were found unstable.

#### Comparative phylogenetic analysis of calcium sensor gene family in finger millet and rice

**Figure 1:**
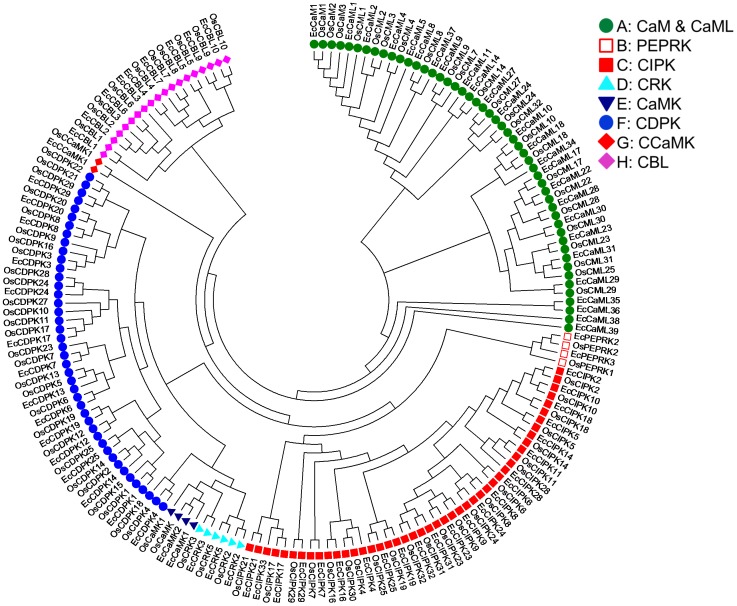
Phylogenetic tree of calcium sensor genes of rice and finger millet. Neighbor-joining tree was created using MEGA6 software with 1000 bootstrap using ORF sequences of rice and finger millet CaM and CaMLs, CBLs, CRKs, PEPRKs, CaMKs and CCaMK proteins. Eight groups were labelled as A, B, C, D, E, F, G and H.

Further, to compare finger millet CaM and CaMLs, PEPRKs, CIPKs, CRKs, CaMKs, CDPKs, CCaMK and CBL with those of rice, a phylogenetic tree was generated using full length protein sequences (**Fig. 1**). Phylogenetic analysis of these genes of rice and finger millet differentiated them into different clusters from A to H. Cluster A contained CaM & CaML sequence, while cluster B & C contained sequences of PEPRK and CIPK respectively. It is also noteworthy that cluster D and E contained both the sequences of CRK and CaMK. Cluster F, G and H contained sequences of CDPKs, CCaMK and CBLs respectively.

### Differential expression analysis based on transcript abundance of calcium sensor genes

**Figure 2:**
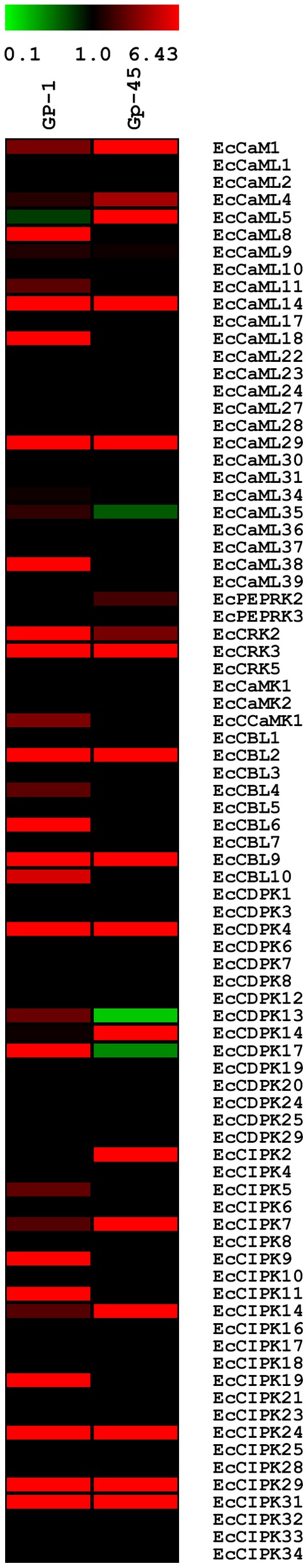
Expression of 82 Calcium sensor genes in pooled spikes of GP-1 (Low calcium) and GP-45 (High calcium) genotype. The number indicated on each cell represents the log2 calculated FPKM values. FPKM values smaller than 1 were not calculated due to negative logarithm and they were stated as in the original data.

In the present study, expression of total 82 calcium sensor genes were compared by using transcriptome data of both GP-1 and GP-45 genotypes. FPKM values are represented in the form of heat map for each identified calcium sensor genes (**Fig. 2**). The results of transcriptome reveals that the expression of 24 genes was higher in the pooled spike sample of GP-45 genotype while the expression of 11 genes was higher in the pooled spike sample of GP-1 genotype. Out of 24 genes highly expressed in the developing spikes of GP-45 genotype, 7 encoded for CaML, 2 for CRK, 5 for CBL, 7 for CIPK and 4 for CDPK genes. Among them, the *EcCIPK9* gene showed very high expression in GP-45 (FPKM value −55.18) when compared to GP-1 genotype (FPKM value −0). The number of genes that were highly expressed in the spike of GP-1 genotype includes 5-CaML, 2-CRK, 3-CIPK, and 1-CDPK genes.

### Pathways analysis of differentially expressed genes

To estimate the functions of the differentially expressed genes of GP-1 and GP-45, biological metabolic pathways were investigated by KEGG pathway analysis. Out of 35 differentially expressed genes, only 10 genes could be assigned to three documented pathways (**Table 4**). These pathways mainly related to stress adaption, hormonal, biotic and abiotic changes. The pathway with the greatest numbers of unique genes was for proteins involved in plant-pathogen interaction. The four genes which have less expression value in GP-45 genotype (*EcCaM1*, *EcCaML4*, *EcCaML14* and *EcCDPK14*) belonged to pathways involved in plant-pathogen interaction and phosphatidylinositol signaling. Out of six genes highly expressed in GP-45 genotype, five (*EcCaML11*, *EcCaML18*, *EcCDPK4*, *EcCDPK13* and *EcCDPK17*) were involved in plant-pathogen interaction and remaining one (*EcCIPK31*) in abiotic stress pathway.

**Table 4:** Functional category of differentially expressed calcium sensor gene in finger millet.

Calcium sensor proteins	Functional pathway
**EcCaM1**	Phosphatidylinositol signaling system, Plant-pathogen interaction
**EcCaML4**	Phosphatidylinositol signaling system, Plant-pathogen interaction
**EcCaML11**	Plant-pathogen interaction
**EcCaML14**	Plant-pathogen interaction
**EcCaML18**	Plant-pathogen interaction
**EcCDPK4**	Plant-pathogen interaction
**EcCDPK13**	Plant-pathogen interaction
**EcCDPK14**	Plant-pathogen interaction
**EcCDPK17**	Plant-pathogen interaction
**EcCIPK31**	Abiotic stresses, Cold stress tolerance

### Quantitative real-time PCR validation

**Figure 3:**
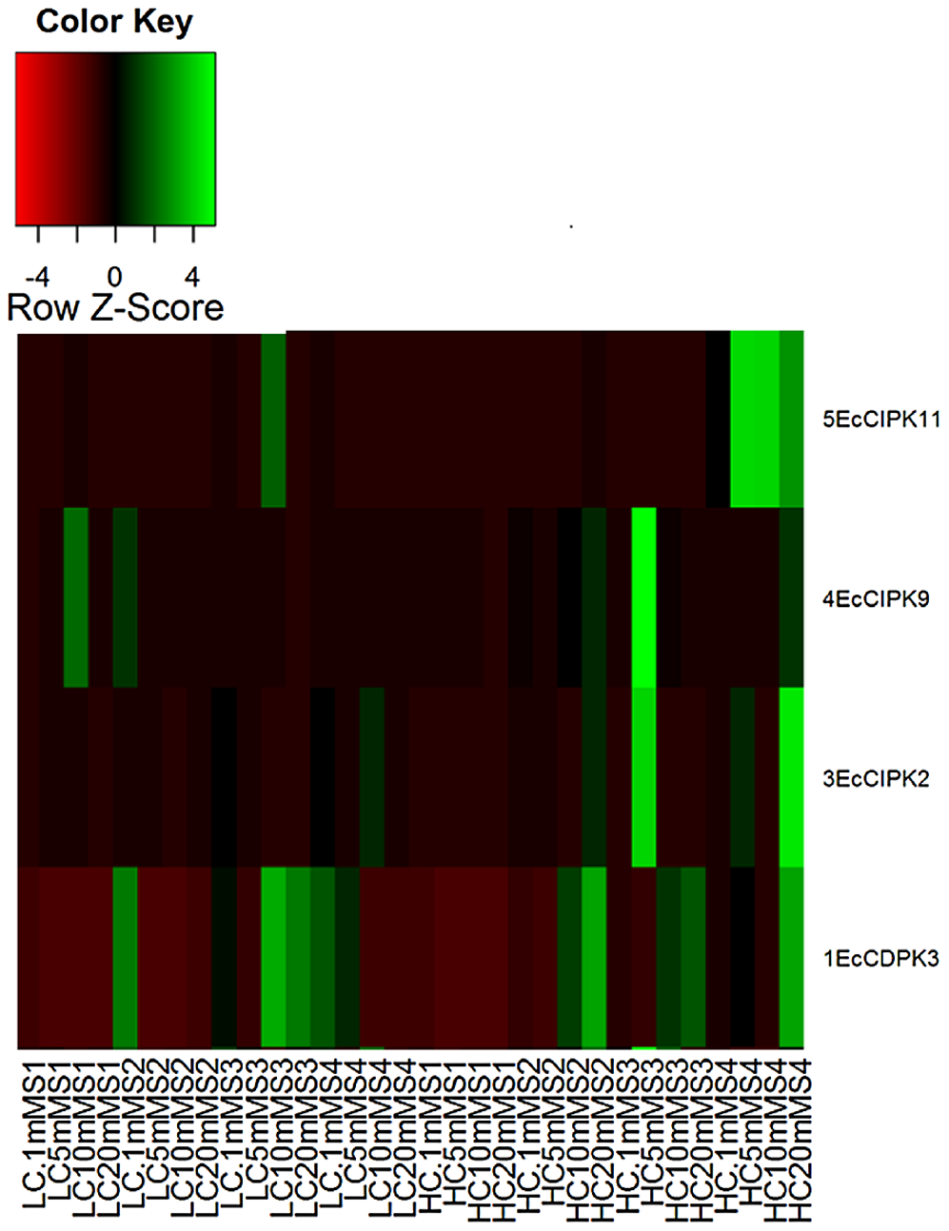
Expression profiles of selected Calcium sensor genes in developing spikes of GP-1 (LC) and GP-45 (HC) exposed to different doses of calcium (0.1 mM, 5.0 mM, 10 mM & 20 mM) as indicated by qPCR analysis. The scale representing the relative signal intensity values is shown above. The different stages of developing spikes are S1 (spike emergence); S2 (pollination stage); S3 (dough stage) and S4 (maturation).

To validate the results obtained from differential expression analysis through RNA sequencing, a total of four calcium responder genes having FPKM more than 10 times higher in GP-45 than in GP-1 genotype, were selected and 4 sets of primers were designed for qPCR study. The details of primer sequences, gene name and Tm are given in **Table 5**. The influence of calcium treatment on the expression of four genes was analysed by the real-time PCR and subsequently a heat map was generated using the R- software package ver. 1.12 (**Fig. 3**). According to heat map, the overall expression of *EcCDPK3*, *EcCIPK2*, *EcCIPK9* and *EcCDPK11* was higher in GP-45 as compared to the GP-1 genotype. The transcript level of *EcCDPK3* gene was comparatively higher in calcium deficient conditions (0.1 mM) in the spike of GP-1 genotype at all four stages. Further increase in concentration of exogenous calcium does not show any marked effect on the expression of the genes in four stages of GP-1 genotype. In GP-45, except for S3 stage at 5 mM, increase in exogenous calcium showed no significant change in the *EcCDPK3* gene expression. Although, a slight increase in the expression was detected in the S2 and S4 stages at 20 mM (**Fig. 3**).

**Table 5:** List of Primers designed from highly expressed genes from transcriptome data of high Ca containing genotype for qPCR analysis.

S.No	Primer code	Primer Seq (5′–3′)	Primer length	Amplicon size	Tm (°C)
1	EcTubulinF	CTCCAAGCTTTCTCCCTCCT	20	207	58
	EcTubulinR	GCATCATCACCTCCTCCAAT	20		
2	EcCDPK3F	ATGTGCGTTCCGTGTACTCC	20	169	60
	EcCDPK3R	ATCTGGATCTCCCTGCGAAT	20		
3	EcCIPK2F	CGATGAGAACAGCAACCTGA	20	152	58
	EcCIPK2R	CCTTTGCACCGTCATAACCT	20		
4	EcCIPK9F	CCGTACGAGCTGGGGAAGA	19	148	61
	EcCIPK9R	CCCGCTTTATCTGCTCGACC	22		
5	EcCIPK11F	AACTTCTGGCTCAGCAGACT	20	157	61
	EcCIPK11R	GGTAAATCGTTCTTCCCGGC	20		

There was no marked effect of exogenous calcium on the expression of *EcCIPK2* in GP-1 genotype while a concentration dependent inducibility was observed in case of GP-45. Sharp induction in expression at S3 and S4 stages and S2 and S4 stages was observed at 5 mM and 20 mM concentration of exogenous calcium, respectively (**Fig. 3**). In case of *EcCIPK9* except at S2 stage (0.1 mM) and S3 stage (10 mM) its expression was almost similar in GP-1 genotype at each concentration of exogenously supplied calcium. In GP-45, the expression of *EcCIPK9* was also similar at each concentration of exogenous calcium supplied; however at S3 stage its expression was exceptionally high at sufficient level (5 mM) of calcium (**Fig. 3**). In case of *EcCIPK11* gene except at S3 stage of GP-1 and S4 stage of GP-45 there was almost no effect of exogenously supplied calcium was observed. In GP-1 at S3 stage almost 305 fold expressions was found at 10 mM (Ca excess). However, at S4 stage of GP-45, concentration dependent increase in the expression of *EcCIPK11* gene was observed (**Fig. 3**).

### Effects of exogenous calcium on its accumulation in developing spikes

**Figure 4:**
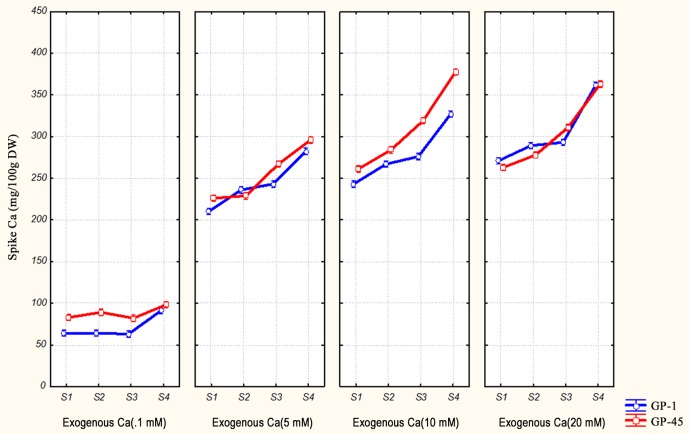
Pattern of calcium accumulation in developing spikes of GP-1 (Low calcium) and GP-45 (High calcium) grown under different concentration of exogenous calcium (0.1 mM, 5.0 mM, 10 mM & 20 mM). The different stages of developing spikes are S1 (spike emergence); S2 (pollination stage); S3 (dough stage) and S4 (maturation).

The data (calcium concentration) obtained from all the samples were used to construct graph by STATISTICA v10.0 (StatSoft, Inc.). The increase of calcium accumulation in the spikes occurred progressively up to 20 mM of exogenously supplied calcium in GP-1 genotype. While in case of GP-45, maximum increase was observed at 10 mM of exogenously supplied calcium (**Fig. 4**). Calcium accumulated significantly (P>0.05) in GP-45 genotype up to 10 mM of exogenously supplied calcium, in comparison to GP-1 genotype which accumulated calcium even at toxic concentration (20 mM). Similar trends of calcium accumulation in the spikes were observed at all four stages of spikes (**Fig. 4**).

## Discussion

This is the most comprehensive study of finger millet transcriptome data till date. GP-1 and GP-45 are the finger millet genotypes varying in the content of seed calcium. The sequence data generated from the transcriptome of both genotypes have been analysed to understand the type and function of calcium sensor gene family by comparative phylogeny and digital gene expression profiling.

### Identification, classification and designation of calcium sensor genes

Identification of any of the gene families in an organism is a very daunting task especially when genome sequence information is not available. In this situation, either domain features of that gene family or sequences characterized from related organisms are used as reference for identification of genes in non-sequenced organisms. In present study, the rice calcium sensor genes were used as query for identifying their homologs in finger millet transcriptome. High level of colinearity of rice genome with finger millet genome [16] and the presence of well characterized sequences of calcium sensor genes in rice are the reasons of its selection as a reference sequence in this study. The calcium sensor genes comprise one of the largest families among plant signaling gene. The member of calcium sensor genes showed varied diversity among the species. In our study, we demonstrated 82 calcium sensor proteins in the transcriptome of finger millet developing spike. The abundance of calcium sensor genes in a species may be related with genome duplications (segmental/tandem), rather than the genome size. Rice genome (0.4 GB) include only (110) sensor proteins [17]–[19]. Further, it is interesting that in some known species (eg. *Arabidopsis thaliana*; genome size ∼125 Mb; Calcium sensor genes 134) [17]–[20] the number of calcium sensor gene family was found to be more than that of large genome size species. However, it should be noticed that the total number of family members can be altered by the type of data source used in the study. Whole genome sequencing studies might be useful to obtain precise protein abundances compared to the transcriptome-wide data sources.

Correct classification of genes into gene families is important for understanding gene function and evolution. Genes of the same family usually share similar sequences, functional domains and even interacting partners. While some gene families are more dynamic in evolution and show species-specific gene members, others are more conserved and found in distantly related species or even across complete kingdoms of life. Gene family classification, *i.e.*, the grouping of genes or proteins into families, often yields important insights into gene function and gene evolution [21]. Many sequence-based methods for automated gene family classification have been developed within the last 20 years.

In present study a total of 82 genes of calcium sensor family identified from transcriptome data were classified that includes 25-CaM & CaML, 9-CRK, 9-CBL, 23-CIPK and 14-CDPK genes. This includes the gene sequences identified from developing spikes of finger millet and may have failed to spot many other genes of this family which are not expressed. Therefore, only phylogenetic approach of computational phylogeny cannot be applied with these gene sequences. Presence of high level of colinearity and sequence similarity between finger millet and rice genomes could be utilised in this situation [16]. The identified finger millet calcium sensors were verified for conserved sequences, motifs and domains alongside maximum sequence identity and comparative phylogeny.

### Structural and functional analysis of calcium sensor genes

#### Domain and motif analysis of calcium sensor genes

Calcium sensor gene family in plants comprises of protein kinase (PKC), EF- hand and NAF- domain [22]–[24]. The CaM and CaM-like gene family in rice contains two pairs of EF-hand domain [25]. However, its number varies in case of finger millet and ranges from one pair of EF- hand to two pair of EF-hand (**Table 3**). The variation in EF hands in CaM and CaML genes generally, related with calcium binding capacity as one EF hand interact with one calcium ion. Accordingly, their function might also be modulated in different biological processes [26]. The gene family PEPRK and most of the CRK comprises of only protein kinase domain in plants. However, in phylogenetic tree the PEPRK and CRK are entirely apart from each other, this is due to variation in the amino acid sequences of these proteins other than at protein kinase region. The rice CaMK, CCaMK and CDPK gene family comprises of protein kinase domain and EF-hand domain [24]. The number of protein kinase domain is one in all three gene family but the number of EF-hand varies from one (as in case of CaMK), two (as in case of CCaMK) and four (as in case of CDPK). Same trend of number of EF-hand was also recorded in case of CaMK and CCaMK of finger millet, but variation was recorded in case of CDPK genes. The wide variability of EF-hand domains proteins shows the diversity of the processes in which Ca^2+^ is involved [26]. The CIPK gene family in rice have no EF-hand, but have kinase and NAF domain [27]. Similarly in finger millet, CIPK consisted of NAF-domain that interacts with Calcenurin B-like sensor proteins (CBLs). Whereas N-terminal part of CIPKs comprises of a conserved catalytic domain typical of Ser-threonine kinases. In plant CBL have same general structure of CaM but they have calcium binding domains with three EF-hand. The finger millet CBL also contains same number of EF-hand. As like similarity in domain feature of calcium sensor proteins of finger millet with their rice orthologs, similarity in motif and conserved sequence analysis of both organisms was also recorded. Similarity in the conserve region of finger millet and rice calcium sensor genes imply their correct identification and classification. Regardless of similarity at conserve region of finger millet with rice calcium sensor genes, variation in number of domains and motifs were also recorded. Variation in number of domain among members of rice and finger millet, calcium sensor genes might be due to incomplete sequence of finger millet genes or due to segmental duplication/deletion during the course of evolution among these genes.

#### Comparative phylogenetic analysis of calcium sensor gene family in finger millet and rice

To classify and predict the biological role of finger millet calcium sensors, phylogenetic distances were computed comparatively with rice calcium sensor proteins (**Fig. 1**). By referring to the rice calcium sensor protein characterization, most of the finger millet calcium sensor proteins were found within same group with rice. However, twelve calcium sensor genes were found diverse from the rice orthologs. This diversity in the structure of some sensor genes of finger millet with rice might contribute some role in the distinctness of calcium content in finger millet and rice.

#### Sub-Cellular localization and stability analysis in calcium sensor genes

Many of the calcium sensor genes location could not be traced because of limitation of information available in the database (**Table 3**). However, an inference can be made from all above experimental analysis that cytoplasm and mitochondria of the cell encase most of the proteins and only a few are present in the secretory pathways. Another parameter studied was of stability and most of the calcium sensor proteins are found unstable according to their instability index parameters. Earlier report also suggests that most of CDPKs proteins are unstable and unstable short-lived protein often comprise regulatory functions [28]. Further wet lab experimentation is needed to be done to verify and validate these *in silico* results.

### Differential expression of calcium sensor genes

Unlike most animals, plants are sessile and cannot migrate from poor-quality environment therefore have developed mechanisms like efficient uptake of minerals in order to adapt to their environment. Thus, they need to tolerate the particular conditions they encounter to survive. This makes plants an ideal system for the study of adaptive variation, and this is particularly true for finger millet, which shows substantial natural variation in terms of mineral accumulation due to genetic variability. The genetic variability in the distribution of minerals in different plants of same species and within the edible tissues has long been thought to be utilized in biofortification strategies [29]. However, the distinctive patterns in the accumulation of minerals in plant tissues, cell types and sub-cellular compartments are the product of selective transport processes catalyzing their short distance as well as long distance movement [30]. As like in finger millet seed which contain different concentration of calcium in its different layers might be the outcome of efficient calcium transport machinery [31].

In general short distance ion movement depends upon the membrane transporters while the long distance ion movement utilizes the xylem and phloem pathways. The movement of most of the mineral elements in phloem fed tissue (like seeds) occurs predominantly through phloem tissue [30]. The immobility of calcium in phloem tissue [32], makes this study very interesting that how calcium could get accumulated in seed. Efforts were made in present investigation to study the role of calcium sensor genes in seed calcium accumulation by differential expression analysis in two selected genotypes.

The Ca^2+^- transporters *viz.*, Ca^2+^-ATPases, Ca^2+^/Cation exchangers and calcium channels are the main class of calcium transporting proteins [33] and calcium sensors *viz.*, CaM & CaML, CBL, CIPK, CDPK, CRK, CaMK, CCaMK and PEPRK are regulatory proteins which play important role in calcium homeostasis [8], [34]. Calcium transporters can directly regulate the trafficking of calcium within cell or tissue while calcium sensors indirectly involved in this process by regulating the activity of calcium transporters [35]. To study the role of calcium sensor gene in seed calcium accumulation, the expression level of these genes were detected by comparing the FPKM value of 82 genes expressed in the spike transcriptome of both the genotypes (**Fig. 2**). FPKM is defined as a quantification method for gene expression by the data obtained from RNA sequencing, to normalize the total read length and the number of sequencing reads [36]. The results of transcriptome reveals that the expression of 24 genes was found higher in the spike of GP-45 genotype and the expression of 11 genes was found higher in the spike of GP-1 genotype. The remaining genes were not assigned any value (0) even though their transcripts were detected. The 0 FPKM values in these genes are due to low expression of these genes and the value smaller than 1 were not calculated due to negative logarithm [37].

In a similar study on different layer of barley leaf transcriptome the transcript of calcium binding and transporter genes was found almost seven times more in calcium rich epidermal cell compared to other cell. Among these were included homologs of the auto-inhibited Ca^2+^-ATPase (ACA) family, the cation exchanger (CAX) family, the annexins and numerous calcium-dependent/calmodulin interacting protein kinases (CDPK/CIPK) and calmodulin [38]. Homologs have also been found to be enriched in the mesophyll (where Ca is high) in the eudicot *Arabidopsis* by transcriptomic [39] and proteomic methods [40]. The tonoplast proteome of Arabidopsis mesophyll cells (the high Ca accumulating cell type in dicots), identified various calcium sensors, Ca^2+^-ATPase, Ca^2+^-Exchanger and two pore calcium channels genes [40]. The abundance of calcium sensor genes in the spike of high calcium containing genotype, as evident from FPKM value as with low calcium containing genotype might be most possible reasons of high seed accumulation in finger millet. The results again indicate the polygenic nature of calcium accumulation, as many genes are differentially expressed in both low and high calcium containing genotypes.

In pathway analysis, out of 35 differentially expressed genes, only 10 can be assigned to three pathways, were detected between GP-1 and GP-45. The majority of the differentially expressed genes belong to pathways involved in plant-pathogen interaction and different kind of biotic and abiotic stresses. During these pathways various calcium mediated signaling are operational. In one of the study it was reported that Arabidopsis AtCIPK24 regulates the activity of vacuolar Ca^2+^/H^+^ exchanger (VCaX1) during salt stress [41]. Similarly, high expression of EcCIPK31 gene in GP-45 and its involvement in salt stress might be linked with activation of any calcium exchanger and in turn high calcium accumulation. However, due to limited knowledge of pathway about differentially expressed gene further study is needed.

### Quantitative real-time PCR validation

The calcium responsive study depicts that the availability of calcium in rhizosphere significantly (P<0.05) increased the calcium content in two genotypes of finger millet (**Fig. 4**). The results clearly revealed differential responsiveness of finger millet genotypes towards exogenously supplied calcium. Through qPCR analysis it was found that the expression of EcCDPK11 gene was significantly higher in high calcium containing genotype (GP-45) than low calcium containing genotype (GP-1) (**Fig. 3**). Its expression was also high at high concentration of exogenous calcium application as well as at later stages of spike development (**Fig. 3**). The higher accumulation of these genes might be associated with high seed calcium accumulation in finger millet by regulating the activity of Ca^2+^- ATPase. Recently, in one of the study co-expression of Ca^2+^-ATPase and CDPK was reported in finger millet [42]. The role of AtCDPK1 in regulation of calmodulin activated Ca^2+^-ATPase2 (ACA2) was well studied in *Arabidopsis thaliana*. In that AtCDPK1 bind at N-terminal auto-inhibitory region of ACA2 and inhibit their activity [8].

The expression of EcCIPK2, EcCIPK9 and EcCIPK11 genes was comparatively higher in high calcium containing genotype (GP-45) in comparison to low calcium containing genotype (GP-1) (**Fig. 3**). Its expression increases as the amount of exogenous calcium in nutrient medium was increased (**Fig. 4**). Comparatively higher expression of these genes was obtained at later stages of seed development. However, the expression of EcCIPK19 gene was not corroborated with the calcium accumulation study and hence it might have no role in seed calcium accumulation. Increased expressions of rice OsCIPK2 gene was reported during K^+^ deficient conditions and suspected that it may regulate activity of the K^+^ channel, enhancing K^+^ uptake under K^+^ -deficient conditions [43]. The role of these genes in high seed calcium accumulation has not yet investigated. In one study, it was demonstrated that Arabidopsis SOS2 (AtCIPK24) regulates the activity of vacuolar Ca^2+^/H^+^ exchanger (VCaX1) [41]. Interaction of CIPK24 with CBL10 at vacuolar membranes was also reported [24] because binding of CBL10 is essential for the full functioning of CIPK24 gene. The higher accumulation of these gene transcripts might be well correlated with the increasing seed calcium content by activating vascular CaX protein in finger millet.

## Conclusions

In this work, we characterize calcium sensor gene families from the transcriptome of developing spikes of two finger millet genotypes differing in their grain calcium contents for ascertaining their role in grain calcium accumulation. To our knowledge, this is first attempt to assemble and characterize the calcium sensor gene family from the developing spike of finger millet transcriptome using Illumina paired-end sequencing methods. Based on the transcriptome assembly, calcium sensor gene family were isolated, classified and characterized. The 82 sequences were predicted as calcium sensor genes in finger millet. Majority of differentially expressed genes (24) among total calcium sensor genes (82) in GP-45 and 11 in GP-1 genotype were found higher FPKM value. Through KEGG pathway analysis out of 35 differentially expressed genes only ten were assigned to stress related pathways. For the validation of the results of transcriptome sequencing, some of the calcium sensor genes (especially responder) having 10 times higher FPKM value in GP-45 than in GP-1 genotype, was used for qPCR analysis under the influence of different concentration of exogenous calcium. Significant correlations with expression value and amount of calcium accumulated were observed. These results fully demonstrate that Illumina paired-end sequencing is a fast and cost effective approach for gene discovery and their characterization.
